# Effect of Manganese Chloride and of Cotreatment with Cadmium Chloride on the In Vitro Proliferative, Motile, and Invasive Behavior of MDA-MB231 Breast Cancer Cells

**DOI:** 10.3390/molecules24071205

**Published:** 2019-03-27

**Authors:** Claudio Luparello

**Affiliations:** Dipartimento di Scienze e Tecnologie Biologiche, Chimiche e Farmaceutiche (STEBICEF), Università di Palermo, 90128 Palermo, Italy; claudio.luparello@unipa.it; Tel.: +39-091-2389-7405

**Keywords:** breast cancer cells, manganese, cadmium, proliferation, chemotaxis, chemoinvasion

## Abstract

We examined the dose–response effect of MnCl_2_ on the proliferative behavior of triple-negative breast cancer MDA-M231 cells vs. immortalized HB2 cells from breast epithelium taken as nontumoral counterparts. We also tested the effect of MnCl_2_ on tumor cell invasiveness in vitro by evaluating the relative invasion indexes through Boyden chamber assays. Moreover, we checked whether cotreatment with both MnCl_2_ and CdCl_2_ could modify the observed biological response by MDA-MB231 cells. Our results show a promotional impact of MnCl_2_ on cell proliferation, with 5 µM concentration inducing the more pronounced increase after 96-h exposure, which is not shared by HB2 cells. Exposure to 5 µM MnCl_2_ induced also an elevation of the relative invasion index of cancer cells. The Mn-mediated stimulatory effects were counteracted by cotreatment with CdCl_2_. These data support the concept that human exposure to high environmental concentrations of Mn may increase the risk of carcinogenesis and metastasis by prompting the expansion and dissemination of triple-negative breast cancer cells. On the other hand, the Mn-counteracting anticancer property of Cd looks promising and deserves a more detailed characterization of the involved intracellular targets aimed to the molecular modeling of specific antineoplastic agents against malignant breast cancer spreading.

## 1. Introduction

Manganese (Mn) is a natural occurring element in the earth’s crust where it represents the twelfth most abundant one. It is not found in nature as a pure metal but as a component of several inorganic and organic compounds. In industry, the major implementations of Mn include steel production and the alloying of aluminum. The major dietary source of Mn for humans is vegetables, e.g., cereals, whole grain, and nuts, and usually Mn intake from food is higher than that from water. However, since the environmental concentration of the metal may be elevated by anthropogenic activities such as industrial emissions, fossil fuel combustion, and use of pesticides, this can result in its release into and contamination of drinking water with consequent increase of human exposure. At cellular level, Mn is an essential micronutrient which can be found as a component of a broad variety of metalloenzymes involved in processes related to energy production, general metabolism, bone and connective tissue formation, and blood clotting [1,2]. Mn uptake by mammalian cells is mediated by different transporters, such as the divalent metal transporter -1 (DMT-1) and Zrt- and Irt-like proteins (ZIP) 8 and 14 [3,4], which show affinity also for other divalent metals, i.e., cadmium (Cd) and zinc (Zn).

Triple-negative breast cancers (TNBC) are characterized by a highly “aggressive” malignant potential thereby being usually associated with worse prognosis than other breast tumor types, due to the lack of expression of estrogen and progesterone receptors and HER2/neu, which renders these neoplastic cells poorly responsive to hormonal therapies and to HER2-targeting drugs [5]. A number of case–control studies have found a significant association between serum Mn levels and the development of malignant breast cancer in humans [6,7] and rodents [8], thus prompting a more detailed biological investigation of the cellular and molecular implications of breast cell exposure to Mn. To this purpose, the MDA-MB231 cell line represents a suitable model system in vitro, being derived from a pleural effusion of a TNBC of basal subtype and displaying several aspects of a highly malignant phenotype, e.g., the inactivation of p53 dependent upon a mutation in codon 280 and the ability to metastasize in vivo [9,10,11]. The number of studies concerning the effect of Mn on MDA-MB231 cells that can be found in the literature is limited. In particular, Lymburner et al. [12] demonstrated that, among other divalent cations, Mn acts as the strongest promoter of integrin-mediated adhesion of MDA-MB231 cells to fibronectin, whereas Ju et al. [13] reported a downregulation of MDA-MB231 cell growth and motility in the presence of Mn-12 acetate.

In the present study we have investigated the dose–response effect of different concentrations of MnCl_2_ on MDA-M231 cell viability/growth comparing cell behavior with that of immortalized HB2 cells from breast epithelium taken as a nontumoral counterpart. We have further examined the effect of MnCl_2_ on TNBC cells with respect to invasiveness in vitro by evaluating specific parameters such as invasion index (I.I.) and relative invasion index (R.I.I.). Moreover, taking into consideration that Mn and Cd share and compete for the same membrane transporters [14,15,16] and that we [17,18,19] previously demonstrated and characterized the cytotoxic effect exerted by exposure of MDA-MB231 cells to CdCl_2_, we checked whether coexposure of MDA-MB231 cells to both MnCl_2_ and CdCl_2_ could somehow modify the biological response of TNBC cells with respect to the treatment with the sole MnCl_2_.

## 2. Results

### 2.1. Influence of MnCl_2_ on Cell Proliferative and Invasive Behavior In Vitro

**Figure 1:**
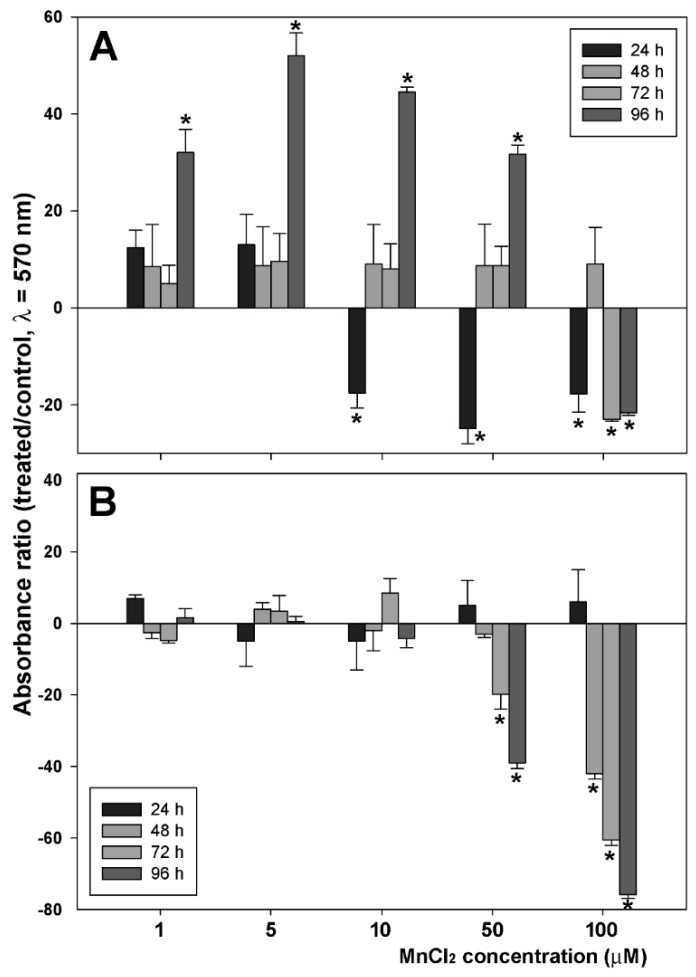
Effect of exposure of MDA-MB231 (**A**) and HB2 (**B**) cells to different concentrations of MnCl_2_ for 24, 48, 72, and 96 h. The histogram shows the percent ratio between the crystal violet absorbance of exposed cells and that of untreated controls. Four replicates were run for each assay. The results are expressed as the mean ± standard error of the mean (SEM) from quadruplicate assays. Four replicates were run for each assay. * *p* < 0.05.

**Figure 2:**
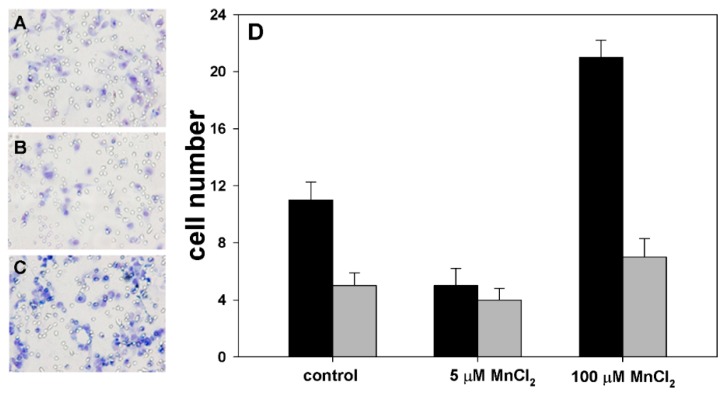
Representative low-magnification images captured at 6-h showing that control, (**A**) 5 µM MnCl_2_-treated (**B**), and 100 µM MnCl_2_-treated cells (**C**) migrated through the filter pores in the chemotaxis assays. Microscopic magnification = 40×. (**D**) Histogram showing the number of migrated cells in chemotaxis (black bars) and chemoinvasion assays (gray bars) in the two experimental conditions. The results are expressed as the mean ± standard error of the mean (SEM) of six different experiments.

In the first set of assays we checked the effect of exposure to increasing concentrations of MnCl_2_ for various time lapses on the proliferative behavior of MDA-MB231 TNBC cells via crystal violet assay. In addition, parallel experiments were performed on HB2, an immortalized cell line obtained from breast epithelium, taken as a non-neoplastic counterpart. Figure 1A shows the dose- and time-response histograms of viability/growth of MDA-MB231 cells as the percentage of dye absorbance in wells with respect to controls. The data obtained indicate that 96-h treatments with 1, 5, 10, and 50 µM MnCl_2_ determined an approximate 32, 52, 47, and 32% increase of cell population, respectively, although an initial decrease of cell number could be observed in the presence of 10 and 50 µM concentrations of the molecule. The dose–time response pattern after exposure to 100 µM MnCl_2_ was symptomatic of a viability- and growth-restraining activity exerted by the molecule at this high concentration on the breast cancer cell line. On the other hand, as shown in Figure 2B, when HB2 cells were submitted to the same analysis for comparison, their proliferative behavior was not remarkably modified by the presence of MnCl_2_, and, also in this case, at the higher concentrations tested, i.e., 50 and 100 µM, the number of attached cells was found to diminish over time.

We then extended our study of MnCl_2_ effects on MDA-MB231 cancer cells by examining their locomotory and invasive behavior in vitro after exposure for 96 h to either 5 or 100 µM concentrations of the molecule, which in the proliferation assays induced higher increase and decrease of the cell population, respectively. When cells were cultured in these two experimental conditions, as shown in Figure 2, an opposite modulation of cell motility in Boyden chamber assays was observed.

In fact, the average migration ratio between either 5- or 100 µM MnCl_2_-exposed cells and controls was 0.45 and 1.9, respectively, in the chemotaxis assays, and 0.8 and 1.4 in the chemoinvasion assays.

In light of the data obtained, we calculated the I.I.s and the R.I.I., a parameter which, taking both cell chemotactic and chemoinvasive behaviors into consideration, is useful to extrapolate the actual modulation of in vitro invasiveness within the general phenomenon of cell mobilization (see e.g., Luparello, C.; et al. [9]). Although the general motile attitude appeared to increase in cells treated with 100 µM MnCl_2_, when I.I.s and R.I.I.s were evaluated, exposure to 5 µM MnCl_2_ appeared to upregulate drastically the ability of the subpopulation of locomotion-triggered cells to penetrate the three-dimensional matrix (control cells’ I.I. = 0.36; treated cells’ I.I. = 1; R.I.I. = 2.78), whereas exposure to 100 µM concentration of the molecule, which promoted the motility of a larger cell population, resulted in a slight downregulation of cell invasive potential (control cells’ I.I. = 0.36; treated cells’ I.I. = 0.33; R.I.I. = 0.92).

### 2.2. Effect of Coexposure to MnCl_2_ and CdCl_2_ on Cell Proliferative and Invasive Behavior In Vitro 

**Figure 3:**
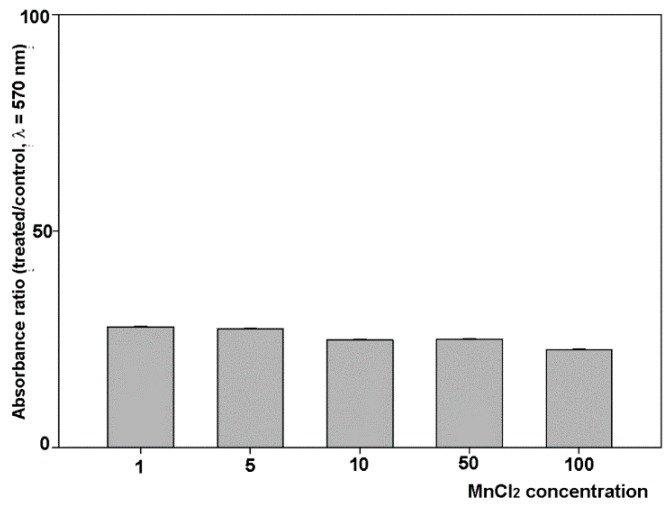
Effect of coexposure of MDA-MB231 cells to both 5 µM CdCl_2_ and different concentrations of MnCl_2_ for 96 h. The histogram shows the percent ratio between the crystal violet absorbance of coexposed cells and that of controls, i.e., cells treated with the sole MnCl_2_ at the indicated concentrations. Results are from quadruplicate crystal violet assays. Four replicates were run for each assay. The results are expressed as the mean ± standard error of the mean (SEM).

We have previously reported that CdCl_2_ is able to restrain MDA-MB231 cell viability and growth with an IC_50_ of 5 µM at 96-h [17]. Therefore, in a second set of experiments we examined the effect of addition of 5 µM CdCl_2_ in the experimental conditions previously tested on MDA-MB231 cell proliferative behavior after 96-h exposure. Figure 3 shows the dose–response histogram as the percentage of dye absorbance in wells with respect to controls, i.e., cells treated with MnCl_2_ only. The data obtained indicate that CdCl_2_ was able to decrease cell growth down to ~22–27% of controls in all the experimental conditions tested, including treatment with 100 µM MnCl_2_ that was the sole condition where no upregulation of cell number was found. Therefore, this result gives evidence that coexposure to CdCl_2_ determines a generalized reversion of MnCl_2_-induced promotion of MDA-MB231 cell proliferative behavior.

**Figure 4:**
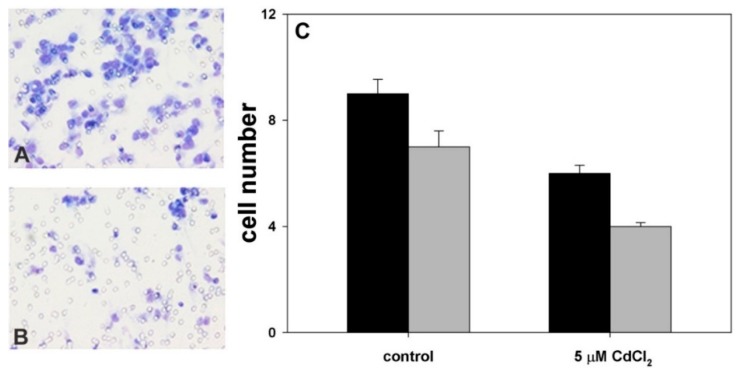
Representative low-magnification images captured at 6 h showing control (**A**) and 5 µM CdCl_2_-treated MDA-MB231 cells (**B**) migrated through the filter pores in chemotaxis assays. Microscopic magnification = 40×. (**C**) Histogram showing the number of migrated cells in chemotaxis (black bars) and chemoinvasion assays (gray bars) in the two experimental conditions. The results are expressed as the mean ± standard error of the mean (SEM) of six different experiments.

The effect of coexposure of MnCl_2_ with 5 µM CdCl_2_ on cell invasive behavior was also checked. Therefore, in preliminary assays we evaluated MDA-MB231 cell behavior in chemotaxis and chemoinvasion assays after exposure for 96 h to 5 µM CdCl_2_. Figure 4 shows that, in line with the previously observed inhibitory effect on cell viability and growth [17], CdCl_2_ treatment determined a decrease of cell chemotactic ability with an average migration ratio between exposed and control cells of 0.66. Analogously, we found a decrease also in cell chemoinvasive ability through three-dimensional reconstituted basement membrane, the average migration ratio accounting for 0.57. The average I.I.s of control and treated cells were found to account for 77 and 67, respectively, and consequently the average R.I.I. of treated cells was 0.87 thus confirming the invasion-restraining effect of cell exposure to CdCl_2_.

**Figure 5:**
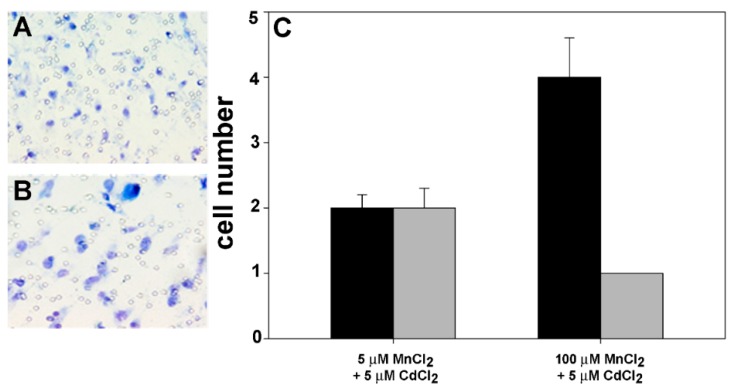
Representative low-magnification images captured at 6 h showing 5 µM MnCl_2_/5 µM CdCl_2_ cotreated (**A**) and 100 µM MnCl_2_/5 µM CdCl_2_ cotreated MDA-MB231 cells (**B**) migrated through the filter pores in chemotaxis assays. Microscopic magnification = 40×. (**C**) Histogram showing the number of migrated cells in chemotaxis (black bars) and chemoinvasion assays (gray bars) in the different experimental conditions. The results are expressed as the mean ± standard error of the mean (SEM) of six different experiments.

In light of the observed stimulatory role played by MnCl_2_ on cell motility or invasiveness in Boyden chamber tests, we examined whether the addition of 5 µM CdCl_2_ in MnCl_2_-containing media could induce any change in the locomotory or invasive attitude of breast cancer cells. Figure 5 shows that CdCl_2_ was able to downregulate the effect of MnCl_2_ on cell invasive potential shown in Figure 2. In particular, the coexposure to 5 µM MnCl_2_ appeared to determine an average migration ratio vs. controls (i.e., cell exposed to 5 µM MnCl_2_ only) of 0.5 in both chemotaxis and chemoinvasion tests, respectively, whereas in the case of cotreatment with 100 µM MnCl_2_, the average migration ratio vs. controls (i.e., cell exposed to 100 µM MnCl_2_ only) decreased down to 0.19 and 0.14 in chemotaxis and chemoinvasion tests, respectively. On the other hand, when the R.I.I.s were quantitated their values appeared to diminish in both experimental conditions of coexposure, being 1 in the presence of 5 µM MnCl_2_ (control cells’ I.I. = 1, co-treated cells’ I.I. = 1) and 0.75 in the presence of 100 µM MnCl_2_ (control cells’ I.I. = 0.33, cotreated cells’ I.I. = 0.25).

## 3. Discussion

In the present paper, we have tested the effect of increasing concentrations of MnCl_2_ on the proliferative, locomotory, and invasive behavior of TNBC MDA-MB231 cells. All the concentrations chosen, including the lowest one, i.e., 1 µM, are higher than those estimated in healthy adults’ physiological fluids such as blood and milk [20,21], and therefore the experimental conditions are representative of states of moderate to intense environmental contamination. Noteworthy, in their early case–control study Mulay et al. [22] demonstrated the increase of Mn concentration in malignant human breast tissues with respect to their normal counterparts, whereas Dearth et al. [8] reported that exposure of prepubertal rats to environmentally relevant high Mn concentrations increases their susceptibility to breast cancer risk via upregulation of ERα and associated proteins. In line with these observations, our first set of data on cell proliferative behavior show a promotional impact of MnCl_2_, except for the highest concentration tested of 100 µM, which is not shared by non-neoplastic HB2 breast cells. Interestingly, in previous experiments HB2 cells resulted insensitive also to the cytotoxic effect of CdCl_2_ when administered at a concentration equivalent to the IC_50_ at 96 h for MDA-MB231 cells [17]. When breast cancer cells were assayed for their motility and invasiveness through matrigel after exposure to the two MnCl_2_ concentrations exerting opposite growth effects after 96 h of incubation, i.e., 5 and 100 µM, they exhibited a stimulation of their motile, but not invasive, attitude in the presence of the higher concentration, whereas those cultured in 5 µM MnCl_2_-containing medium appeared endowed also with a marked upregulation of their invasive, other than proliferative, phenotype. It is known that, in addition to the activation of the locomotory ability, highly invasive MDA-MB231 subpopulations selected using Boyden chambers display (i) the upregulation of urokinase-plasminogen activator (uPa)/uPa receptor expression and of metalloproteinase (MMP) activity, and (ii) an epithelial–mesenchymal transition leading to a weaker attachment to extracellular matrix (ECM) molecules (such as matrigel components) due to the downregulation of genes involved in cell–ECM adhesion and cell–cell junctions [23]. Both aspects support cell infiltration and crossing through the three-dimensional matrigel layer.

Among the enzymes that use Mn as a cofactor, manganese superoxide dismutase (MnSOD) is a likely candidate as responsible of the observed effect. MnSOD is a mitochondrial tetrameric protein contributing to the maintenance of redox balance and intact respiratory function via the dismutation of the superoxide radical to oxygen and hydrogen peroxide, the latter being subsequently metabolized by hydrogen peroxide-detoxifying enzymes [24]. High levels of MnSOD gene (i.e., *SOD2*) expression have been found in different cancers and correlated to poor prognosis, low relapse-free survival and overall survival rate. Concerning breast cancer, *SOD2* expression levels are different in the various histotypes, being upregulated in both tissue specimens and cell lines derived from the more “aggressive” basal-like, claudin low-, and estrogen receptor-negative subtype, including MDA-MB231 [25]. Kattan et al. [26] reported that the elevated expression level of *SOD2* in MDA-MB231 cells is coupled to a downregulation of the detoxifying enzymes, thereby resulting in an accumulation of hydrogen peroxide, which, in turn, stimulates cell growth via activation of the MAPK-dependent signalization and transcription factors such as NF-κB and AP-1. Conversely, low levels of MnSOD protein were found in nontumoral breast epithelial cell lines, such as MCF-10A [27]. A number of experimental evidence has demonstrated that, apart from upregulating MnSOD activity, Mn promotes *SOD2* expression in different biological systems [28,29], including Hs578T TNBC cells [30]. Therefore, it is conceivable that exposure of MDA-MB231 cells to MnCl_2_ may determine an additional dose- and time-dependent upregulation of MnSOD with consequent increase of cell number. Moreover, by analogy with Hs578T line which was induced to death by MnCl_2_ above 100 µM, such concentration impaired also MDA-MB231 cell viability and proliferation. As hypothesized by Thongphasuk et al. [30], such an effect could be due to excess of free radicals and/or high glucose consumption during the redox cycle to supply reducing equivalents. On the other hand, similarly to what was found for the syngeneic Hs578Bst cell line derived from adjacent normal tissue, no effect was exerted by MnCl_2_ treatment on nontumoral HB2 cells, apart from the intoxication at high concentration of the compound as for tumor cells. Such insensitivity might be explained in light of (i) the demonstrated heterogeneity of cell-specific *SOD2* stimulants resulting in the inability of Mn to upregulate the enzymatic activity in certain cytotypes, (ii) the low levels of MnSOD enzyme which may be saturated at nanomolar concentrations of Mn without further dose-dependent stimulation of enzyme function, as reported by [28], and/or (iii) the presence of a more efficient hydrogen peroxide-detoxifying apparatus [30].

The involvement of MnSOD in cell invasive activity is also widely recognized. Nelson et al. [31] demonstrated the association between the elevated MnSOD enzymatic activity and the upregulation of general MMP expression in HT-1080 fibrosarcoma cells via the hydrogen peroxide-triggered increase of the DNA-binding activity of specific transcription factors. The migratory phenotype of these tumor cells, linked to cytoskeletal reorganization, lamellipodia extension, and disruption of focal adhesions, have been also associated to hydrogen peroxide-mediated modulation of Rac and Rho-mediated signal transduction [32]. Concerning MDA-MB231 cells, MnSOD expression was found positively correlated to the epithelial–mesenchymal transition responsible of their invasive and migratory capacities [33]. In line with literature data, the present results demonstrate that MDA-MB231 cells show an approximately threefold increase of their R.I.I. in the presence of 5 µM MnCl_2_, which is attributable to an elevation of motile activity and erosive capacity towards matrigel. On the other hand, the results obtained with cells cultured in 100 µM MnCl_2_-containing medium indicate that, although the R.I.I. underwent a faint lowering, tumor cells still retain an active motile behavior on matrigel-free filters, even greater than controls, thereby suggesting that Mn regulation of viability/growth rate, locomotory stimulation, and release of ECM-targeted enzymes may rely upon different intracellular pathways and mechanisms.

Cd is a nonessential element with a long biological half-life of ~25 years in the human body. It is not involved in enzymatic activities but it was shown to interfere with zinc-dependent intracellular processes, due to their many chemical similarities. Several literature reports have brought evidence about the inhibitory potential of Mn supplementation on intracellular Cd uptake thereby playing a major role in tissue protection from Cd toxicity. Competition for both binding sites at the plasma membrane and transmembrane transport systems, and inhibition of Cd-induced reactive oxygen species (ROS) production, lipid peroxidation, and ERK activation are considered as the main reasons for the Mn-dependent alleviation of Cd effect [3,4,14,15,16,34,35,36]. Concerning TNBC MDA-MB231 cells, our previous data demonstrated the dose-dependent cytotoxic effect of CdCl_2_ with an IC_50_ at 96 h of 5 µM concentration. Subsequent molecular studies demonstrated that this treatment induced the upregulation of cellular defense genes such as those coding for Dap kinase and some members of cytosolic/mitochondrial heat shock proteins, caspases, and metallothioneins, and the downregulation of the genes coding for the survival factor Bcl-2, the NF-κB-stimulating AEG-1 protein, and the cytochrome oxidase subunits II and IV. Moreover, an abnormally increased rate of mitochondrial respiration, a massive accumulation of ROS and a derangement of MAPK pattern of expression and synthesis was also found in exposed cells [17,18,19,37]. To our knowledge, here we present first data focusing on the mitigating activity of Cd on Mn-induced exacerbation of TNBC cell proliferative and invasive behavior. In fact, the addition of 5 µM CdCl_2_ to the medium containing the different MnCl_2_ concentrations counteracted the increase of cell number diminishing cell viability/growth rate down to values close to those attributable to the effect of the sole CdCl_2_. Also, the R.I.I. of coexposed cells decreased drastically with respect to controls. Given that MnSOD is strictly localized into the mitochondria and that the MAPK signaling pathway is responsible of the Mn-triggered proliferative activity [26], it is conceivable that the previously observed alterations induced by Cd to mitochondria and MAPK signalization in MDA-MB231 cells may be, at least in part, responsible of the suppression of the promoting effects of MnCl_2_ in the case of cotreatment. Other non-MnSOD-dependent mechanisms may be responsible of the observed effects. In fact, it must be taken into consideration that Cd and Mn share a common pathway for entering the cells through transmembrane transport systems and that competition between the two metal has been reported [3,4]. In addition, data have been produced on the competitive inhibition played by Cd on Mn-binding active sites in different enzymes and other intracellular targets [34,35,38].

## 4. Materials and Methods

### 4.1. Cell Cultures

MDA-MB231 TNBC cells and HB2 immortalized breast epithelial cells [39] (the latter courtesy of Cancer Research, UK), taken from laboratory stocks, were cultured in RPMI 1640 medium (MDA-MB231) and high glucose–DMEM medium plus 5 µg hydrocortisone/mL and 10 µg bovine insulin/mL (HB2), respectively, both purchased from Sigma, St. Louis, MO, USA, supplemented with 10% fetal calf serum (FCS; ThermoFisher, Waltham MA, USA) and antibiotic/antimycotic mixture (100 U/mL penicillin, 100 µg/mL streptomycin, and 2.5 mg/L amphotericin B; ThermoFisher), at 37 °C in a 5% CO_2_ atmosphere. Since the only Mn source in routine culture is FCS where the metal is present in varying concentrations, its percentage in the tumor cell-containing media was lowered to 1% 4 h before the proliferation assays and maintained at this concentration during the tests. On the other hand, no reduction of serum concentration was made for HB2 cells due to the demonstrated strong dependency of these cells on 10% FCS supplement for their sustained growth rate [40].

### 4.2. Proliferation Assay

Cell proliferative behavior was evaluated by crystal violet assay [41]. Cells in exponential growth were plated at a concentration of 5500 cells/well in a 96-well plate, allowed to adhere overnight, and then treated for 24, 48, 72, and 96 h with 1, 5, 10, 50, or 100 µM MnCl_2_. A parallel set of assays was made by exposing cells to the above-mentioned concentrations of MnCl_2_ in association with 5 µM CdCl_2_. At the end of the incubation cells were stained with 0.2% crystal violet in 2% ethanol for 10 min, followed by elution of the bound dye with 1% SDS. The absorbance of the dissolved crystal violet was measured in an automated microplate reader at 570 nm.

### 4.3. In Vitro Chemotaxis and Chemoinvasion Assays

Cell motile and invasive behaviors were evaluated by a modified Boyden chamber test, essentially as described by [9]. Each “blind well” chamber (top well = 800 µL, bottom well = 200 µL; Neuro-Probe, Cabin John, MD, USA) accommodated a polyvinylpyrrolidone-free filter polycarbonate filter with an 8-µM pore diameter and 50 mm^2^ exposed area (Nucleopore, Pleasanton, CA/USA).

For chemotaxis assays, in order to optimize cell adhesion, the plastic filters were coated with type I collagen (Sigma, St. Louis, MO, USA) dissolved in 0.1% acetic acid at the concentration of 20 µg/mL and allowed to dry overnight [42]. For chemoinvasion assays, the filters were coated with 25 µg of matrigel, a reconstituted three-dimensional basement membrane matrix from Engelbreth–Holm–Swarm murine sarcoma (Sigma, St. Louis, MO, USA).

Before the assays, the cells were treated with MnCl_2_, CdCl_2_, or both, at the concentrations and for the times which were shown to affect their proliferative behavior, as from the previous assay. Trypsinized control and treated cells were resuspended in FCS-deprived medium after exhaustive washing and seeded at the concentration of 3 × 10^5^ in the upper well of the chamber, whereas fresh 10% FCS-containing medium was used as chemoattractant in the lower well, as reported by Kamath and coworkers for MDA-MB231 and MCF-7 breast cancer cells [43]. The assays were carried out for 6 h, and after removal of the nonmigrated cells attached to the upper surface of the filters with a cotton swab, those migrated to the lower surface of the filter were fixed with 95% ethanol, stained with 0.02 g toluidine blue/mL, and quantified by counting their number in twelve random fields of the filter at 200-fold magnification, after mounting the filters onto microscope slides with a drop of immersion oil.

The I.I. and the R.I.I. were calculated as follows

I.I. = N_CI_/N_CT_ × 100

where N_CI_ and N_CT_ were the number of control or treated cells crossing the filters in parallel chemoinvasion and chemotaxis assays, respectively.

R.I.I. = I.I._t_/I.I._c_

where I.I._t_ and I.I._c_ were the I.I. of treated and control cells, respectively. R.I.I. values = 1, <1, and >1 are indicative of invasion-ineffective, -restraining, and -promoting effects exerted by the specific treatment, respectively.

### 4.4. Statistics

Statistics was checked through ANOVA test with SigmaStat 4.0 software (SYSTAT, San Jose, CA, USA). A *p*-value < 0.05 was considered statistically significant.

## 5. Conclusions

Although caution must be exercised in extrapolation of in vitro results to the in vivo situation, the present data further support the concept that human exposure to high environmental concentrations of Mn may increase the risk of accelerating the processes of carcinogenesis and metastasis by prompting the expansion and dissemination of subpopulations of overtly transformed TNBC cells, in line with the findings described in a number of epidemiological reports [7,44,45]. Interestingly, CdCl_2_ treatment appears to possess the potential to counteract MnCl_2_-mediated cancer-promoting effects. This might be achieved by cotargeting cellular sites and components, and reprogramming the molecular and biochemical pathways of MnCl_2_-stimulated TNBC cells. The inhibitory activity of Cd-containing compounds on viability and growth of normal and neoplastic cells and the mitigatory effect provided by Zn-containing molecules have been reported in the literature [46,47,48]. Taking the limited treatment options against TNBC into account, the specific anticancer modulatory property of CdCl_2_ towards MDA-MB231 cells here reported looks promising and deserves a more detailed biological characterization of the involved intracellular targets aimed to the possible molecular modeling of specific antineoplastic agents with improved effectiveness towards the inhibition of the spreading of malignant breast cancer.
